# Numerical Simulation of the Behavior of Toroidal and Spheroidal Multicellular Aggregates in Microfluidic Devices with Microwell and U-Shaped Barrier

**DOI:** 10.3390/mi8120358

**Published:** 2017-12-11

**Authors:** Maryam Barisam, Mohammad Said Saidi, Navid Kashaninejad, Raja Vadivelu, Nam-Trung Nguyen

**Affiliations:** 1 Department of Mechanical Engineering, Sharif University of Technology, Tehran 11155, Iran; Barisam.m@gmail.com; 2 Queensland Micro- and Nanotechnology Centre, Nathan Campus, Griffith University, 170 Kessels Road, Brisbane, QLD 4111, Australia; n.kashaninejad@griffith.edu.au (N.K.); raja.vadivelu@griffithuni.edu.au (R.V.)

**Keywords:** numerical simulation, microfluidics, oxygen/glucose distribution, shear stress, multicellular aggregates, toroid, spheroid, U-shaped barrier, microwell, bioreactor

## Abstract

A microfluidic system provides an excellent platform for cellular studies. Most importantly, a three-dimensional (3D) cell culture model reconstructs more accurately the in vivo microenvironment of tissue. Accordingly, microfluidic 3D cell culture devices could be ideal candidates for in vitro cell culture platforms. In this paper, two types of 3D cellular aggregates, i.e., toroid and spheroid, are numerically studied. The studies are carried out for microfluidic systems containing U-shaped barrier as well as microwell structure. For the first time, we obtain oxygen and glucose concentration distributions inside a toroid aggregate as well as the shear stress on its surface and compare its performance with a spheroid aggregate of the same volume. In particular, we obtain the oxygen concentration distributions in three areas, namely, oxygen-permeable layer, multicellular aggregates and culture medium. Further, glucose concentration distributions in two regions of multicellular aggregates and culture medium are investigated. The results show that the levels of oxygen and glucose in the system containing U-shaped barriers are far more than those in the system containing microwells. Therefore, to achieve high levels of oxygen and nutrients, a system with U-shaped barriers is more suited than the conventional traps, but the choice between toroid and spheroid depends on their volume and orientation. The results indicate that higher oxygen and glucose concentrations can be achieved in spheroid with a small volume as well as in horizontal toroid with a large volume. The vertical toroid has the highest levels of oxygen and glucose concentration while the surface shear stress on its surface is also maximum. These findings can be used as guidelines for designing an optimum 3D microfluidic bioreactor based on the desired levels of oxygen, glucose and shear stress distributions.

## 1. Introduction

Three-dimensional (3D) multicellular aggregates with the capability to mimic cell-cell and cell-matrix interactions can bridge the gap between two-dimensional (2D) monolayer cultures and animal models. On the other hand, microfluidic platforms can produce spatial and temporal gradients and can mimic the dynamics of tissue microenvironment [1,2]. The use of a microfluidic system [3,4,5,6,7] to culture 3D multicellular aggregates presents a precise and suitable model for cellular studies [8]. 

Spheroids are well-characterised cell models for 3D cell cultures that are created by self-assembling of cells with or without scaffolding [9,10,11]. However, as the diameter of spheroid increases, diffusions of oxygen, nutrients and metabolic waste become limited. This reduction of diffusion rate may lead to cell death and the creation of necrotic areas [9,10]. A solution for this issue is using a doughnut-shaped 3D multicellular aggregate called toroid. Toroidal cell culture can enhance the exchange of oxygen and nutrients (e.g., glucose) and accordingly allow for a long-term in vitro cell culture.

**Figure 1:**
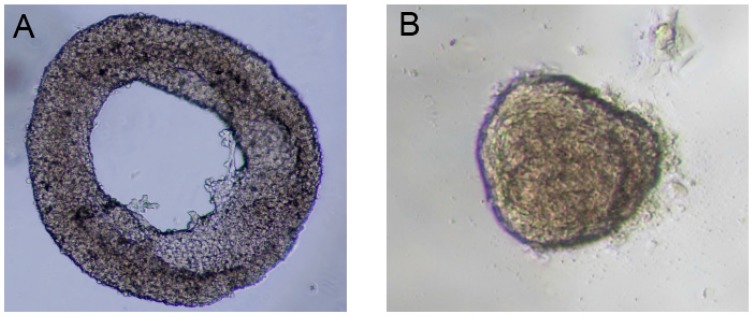
(**A**) Three-dimensional (3D) toroidal multicellular aggregate of fibroblast; (**B**) 3D spheroid multicellular aggregate of fibroblast. The cell densities are 5000 cells/µL and 500 cells/µL for toroid and spheroid, respectively. The cells are grown with an agarose hydrogel containing 20% fetal bovine serum (FBS).

Various methods such as hanging-drop method, mechanical trapping and force-driven methods have been devised for the production of spheroids [12,13]. However, the methods for toroid production are still under development. Masuda et al. [10] used microwells with central microposts to create toroidal multicellular aggregates with a micro-lumen due to non-adherence nature of the walls. They demonstrated that cell viability for toroid was higher than that of the spheroid. Chumtong et al. [14] used a similar system to produce the toroid. Their system had an array of deformable scaffolds coated by an elastic membrane with a central micropillar. Takai et al. [15] developed a microfluidic device that generated toroid aggregate by creating a rotational flow. Recently, our group introduced a new technique for multicellular toroid fabrication [16]. First, agarose hydrogel was prepared by mixing 20% fetal bovine serum (FBS) to make the final concentration 0.5%. After that, the droplet was dispensed on polytetrafluoroethylene (PTFE) powder for 2 min to solidify. Then, cells with a density of 5000 cells/µL were diluted in the culture medium (Dulbecco’s Modified Eagle Medium). Subsequently, 10 µL of the cell suspension was placed on the solidified gel to obtain a droplet containing agarose and cells. This composite droplet was then coated with the hydrophobic powder (PTFE) to sustain its spherical shape (i.e., the standard way to form liquid marble). The cells were induced to migrate and accordingly formed the toroid aggregates. In the present paper, we followed the same procedure with different cell types (fibroblast cells) to produce both toroidal (Figure 1A) and spheroidal (Figure 1B) multicellular aggregates. 

In previous publications, we discussed the effect of cell density on spheroid [17] and toroid [16]. In summary, to fabricate toroidal aggregate, cell density should be large enough (i.e., 5000 cells/µL) while for spheroid formation, the cell density needs to be much smaller (i.e., 500 cells/µL). This difference in cell density has been illustrated in Figure 1 for fibroblast cells. As a result, it is not experimentally possible to systematically compare the performance of toroid and spheroid aggregates under different flow and oxygen concentration. The second challenge is controlling the orientation of a toroid in a microfluidic system with a U-shaped barrier. Multicellular aggregates are first formed in a liquid marble (i.e., outside of a microfluidic system). These toroid or spheroid aggregates are then injected to the microfluidic system by hydrostatic pressure and will be trapped in the designated areas. Toroid can be trapped both in horizontal and vertical orientations in the microfluidic system with a U-shaped barrier. Vertical or horizontal orientation of toroid is relative to medium flow direction. Thus, with the current U-shaped barrier considered in the literature, controlling the orientation of the toroid is not possible. Accordingly, numerical simulation with finite element method to discretise the governing equations of fluid flow and concentration can be a versatile approach. 

In spheroid cell culture, numerical simulations had been successfully conducted to obtain the oxygen and glucose concentration distributions. The authors investigated the effect of various parameters such as spheroid diameter [18,19,20], wall permeability [21,22] and inlet concentration [21,22,23] on the concentration distribution in the spheroid. Astolfi et al. [22] presented a 3D numerical model considering the wall permeability and concentration jump (the difference in the concentration of interface between two regions due to the difference in solvability) between the two regions, i.e., tissue-culture medium and polydimethylsiloxane (PDMS)-culture medium. However, to the best of our knowledge, oxygen and glucose concentration distributions inside the toroid are not investigated and compared to those inside the spheroid.

In this research, oxygen and glucose concentration distributions inside toroid and spheroid are investigated numerically. We compared the performance of two commonly used microfluidic platforms to trap and culture 3D multicellular aggregates, namely, a microfluidic device with a U-shaped barrier [24,25,26] and the one with microwell structures [27,28,29] by solving continuity, momentum and convection-diffusion equations. Accordingly, the concentration distributions of oxygen and glucose under various conditions are examined and compared. In these simulations, the wall permeability, the concentration jump between the regions and the non-linear reaction inside the aggregates are also considered.

## 2. Materials and Methods 

### 2.1. Geometry

**Figure 2:**
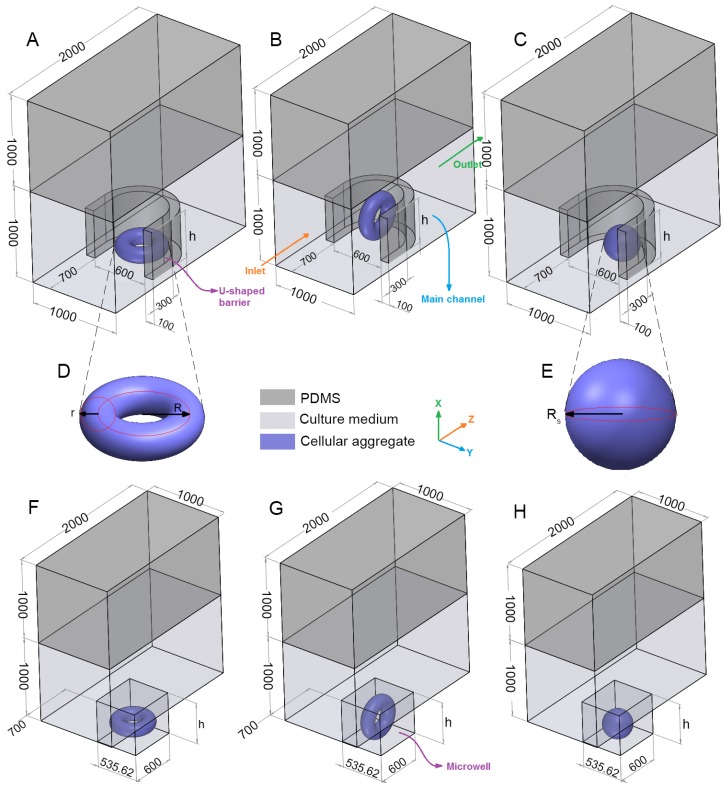
Geometry of the problem (dimensions are in μm) (**A**) horizontal toroid in a U-shaped barrier; (**B**) vertical toroid in a U-shaped barrie; (**C**) spheroid in a U-shaped barrie; (**D**) toroi; (**E**) spheroi; (**F**) horizontal toroid in a microwel; (**G**) vertical toroid in a microwell and (**H**) spheroid in a microwell.

The geometry of the problem consists of a rectangular channel with an oxygen-permeable upper wall (e.g., fabricated from PDMS), an impermeable lower wall (e.g., it can be made from PDMS but must be placed on an oxygen-impermeable plate when being tested in the lab) and a row of arrays of traps (Figure 2A–C,F–H). Considering the fabrication technology, the whole device including the lower layer needs to be fabricated from the same materials (e.g., PDMS, 3D printing resin, etc.). However, when tested in lab, it needs to be placed on a platform that is most likely impermeable to oxygen. Due to the symmetry of the problem, just one trap is considered and symmetry boundary condition is applied to the side surfaces. The trap is either in the form of a rectangular microwell or a U-shaped barrier (made from PDMS). Both types of the traps have equal height (*h*), length (600 μm) and cross-section area (≅0.321 ${\mathrm{mm}}^{2}$). The center of the traps, which is located at the half of the trap length and width are placed at the center of the lower surface of the main channel. 

Two types of multicellular aggregates (Figure 2D,E), one in the form of a spheroid and the other in the shape of a toroid are considered and investigated. Two orientations for the toroid are examined, i.e., horizontal and vertical shapes with the centerline axis parallel to *x* and *y*, respectively. The orientations are shown in Figure 2A,B,F,G. To remove the mesh computational error, particularly in the case of vertical toroid, there should be a margin distance between the multicellular aggregate and the trap walls. We chose this margin distance to be 1/1000 of the channel width, i.e., 1 μm. A margin distance less than this value would substantially increase the computational time. We observed that there was no significant difference between the results when the margin distance was either zero or one micron. For example, for a horizontal toroid with the minor radius *r* = 80 μm, and the major radius *R =* 160 μm in a U-shaped barrier with the trap height *h =* 600 μm, we compared the effects of margin distance on the average oxygen concentration ($c_{ave,O_{2}}$), the average glucose concentration ($c_{ave,glucose}$) and maximum shear stress (***τ_max_***) in Table 1. Due to the difference in wettability and material properties of the channel and the aggregates, this margin distance is expected in experiment as well. 

### 2.2. Governing Equations

Since the characteristic dimension of the device (*L*) is much larger than the mean free path length of the liquid flowing in the device (*λ*), the fluid is considered to be continuum. This can be illustrated by Knudsen number, Kn=λ/L, which should be less than 0.001 so that the continuity equation can be applied. In the present device, *L* = 80 μm and *λ* ≅ 0.25 nm (the mean free path of water) so the $Kn=3.125\times 10^{-6}$. Accordingly, it is assumed that incompressible and steady laminar flow of culture medium continuously flows inside the channel and around the aggregate. The physics of the fluid flow in this condition is governed by a simplified form of continuity and momentum equations as follows:

(1) $$\vec{𝛻}\cdot \vec{V}=0$$

(2) $$\rho \left(\vec{V}\cdot \vec{𝛻}\right)\vec{V}=-\vec{𝛻}p+\mu {𝛻}^{2}\vec{V}$$

where, $\vec{V}$ is velocity vector with *x*, *y* and *z* components, $\vec{𝛻}p$ is the pressure gradient, ρ and μ are respectively the fluid density and viscosity. $\vec{𝛻}$ is the del operator in vector calculus and accordingly ${𝛻}^{2}$ represents Laplacian and $\vec{𝛻}$ shows the divergence of a vector. The velocity inside the cellular aggregate was considered to be zero, and its outer surface was modelled as a wall for medium flow.

To estimate the concentration distribution of oxygen and glucose, the general form of convection-diffusion equation needs to be solved [30]:

(3) $$\frac{\partial C}{\partial t}+\vec{V}\cdot \vec{𝛻}C=\vec{𝛻}\cdot \left(D\vec{𝛻}C\right)-R$$

where C is the concentration of the species and D is diffusion coefficient. $\frac{\partial C}{\partial t}$ is the transient variation of concentration as a function of time. In this study, this transient term is not considered because the boundary conditions, the reaction rate and the fluid flow are all in steady-state conditions. The term $\vec{V}\cdot \vec{𝛻}C$ is the convetion term which indicates the change of concentration as a result of bulk fluid motion. This term is important to find the distributions of oxygen and glucose in culture medium region. The term $\vec{𝛻}\cdot \left(D\vec{𝛻}C\right)$ shows the concentration gradient due to the molecular diffusion. For most cases, diffusion coefficient is constant, and this term simplifies to $D{𝛻}^{2}C$. Finally, R is the reaction rate which describes how the quantity of interest (here, oxygen or glucose) is produced or consumed in a particular region as a result of chemical reaction. 

In this study, we first calculated the available concentrations of oxygen and glucose around the cellular aggregate. Oxygen is available to the tissue from two regions: dissolved oxygen in the culture medium flow and the ambient oxygen which diffuses through the PDMS layer. Glucose is only availabe from the culture medium flow. Assuming a steady-state condtion with constant diffusion coefficient and zero reaction rate, Equation (3) can be simplified as follows:

(3a) $$\vec{V}\cdot \vec{𝛻}C=D{𝛻}^{2}C$$

This steady-state convection-diffusion equation (Equation (3a)) was coupled with continuity and momentuum equations (Equations (1) and (2)) to find the glucose and oxygen concentration distributions in the culture medium flow. To find the oxygen concentration in the PDMS layers, pure diffusion equation (Equation (3b)) was solved in the corresponding domain:

(3b) $${𝛻}^{2}C=0$$

Finally, to find the oxygen and glucose concentrations inside the tissue (i.e., the cellular aggregates in the form of toroid and spheroid), the pure diffusion equation with a reaction rate, Equation (3c), was solved in the tissue region (the equation was solved sperately for oxygen and glucose with the corresponding values):

(3c) $$D{𝛻}^{2}C=R$$

To take into account the oxygen and glucose consumption rates by the cells, Equation (3c) was coupled with Michaelis–Menten reaction ([18,20,22,31,32]) term (Equation (4)):

(4) $$R=\frac{V_{max}C}{C+K_{m}}$$

where $V_{max}$ and $K_{m}$ are the maximum reaction rate and Michaelis constant, respectively. All the required parameters are listed in Table 2. The culture medium properties are assumed to be similar to those of water at the desired temperature (37 °C).

### 2.3. Boundary Conditions

Fully developed velocity profile compatible with constant flow rate (Q = 5$\frac{\mu L}{\min}$) is applied at the inlet. Zero pressure, symmetry boundary condition and no-slip boundary condition are applied at the outlet, side surfaces (the surfaces parallel to *x*-*z* plane) of the main channel and the other surfaces in contact with the culture medium, respectively. The apparent slip length, ${ℒ}_{s}$, on most hydrophobic walls is less than 100 nm [33]. This slip length is scaled based on the device characteristic dimension L, and the ratio of ${ℒ}_{s}/L$ determines the relative importance of slip boundary condition. Thus, for liquid flow at $Kn\ll 10^{-3}$, even on hydrophobic walls, the assumption of no-slip boundary condition holds true [33]. In other words, it can be assumed that the velocity of the fluid flow in the vicinity of a stationary wall is equal to that of the wall, i.e., zero. Therefore, no-slip boundary condition was used for all the walls including the interface of the aggregate and culture medium. In symmetry boundary condition, the mass flow rate and any scalar flux across the boundary are considered to be zero.

For the convection–diffusion equation, concentration inflow ($c_{0,O_{2}}$ = 0.2 mM and $c_{0,glucose}$ = 11 mM) and outflow are considered for the inlet and outlet, respectively. To obtain the oxygen concentration, symmetry boundary condition is applied at the main channel as well as the side surfaces of the PDMS layer. However, this boundary condition was only applied at the main channel for glucose. To take into account the concentration jump, constant concentration proportional to inflow concentration is applied ($S_{O_{2}-PDMS\;vs\;H_{2}O}\times c_{0,O_{2}}$) at the top surface of the PDMS layer. At the interface of the culture medium, the PDMS layer and tumor aggregate, equal flux and concentration jump are considered. That is, at the interface, $c_{O_{2},PDMS}=S_{O_{2}-PDMS\;vs\;H_{2}O}\times c_{O_{2},medium}$, $c_{O_{2},aggregate}=S_{O_{2}-CT\;vs\;H_{2}O}\times c_{O_{2},medium}$ and $c_{glucose,aggregate}=S_{glucose-CT\;vs\;H_{2}O}\times c_{glucose,medium}$. It should be noted that to find the glucose concentration distribution, PDMS domain is not included due to its impermeability to glucose. No-flux is applied to the other surfaces.

### 2.4. Numerical Method

The unstructured tetrahedral grid is generated using Tessellation method to mesh all domains and mesh refinement is applied to the interface surfaces and the boundaries. Minimum element size was set to 0.5 μm (in cell aggregate) and 5 μm in all other interfaces. Using finite element approach, the governing equations and the boundary conditions are discretized. To find an approximate solution, residual value less than $10^{-6}$ for continuity and momentum equations and $10^{-3}$ for convection-diffusion equations are used as convergence criteria.

### 2.5. Grid Independency Study and Validation

**Figure 3:**
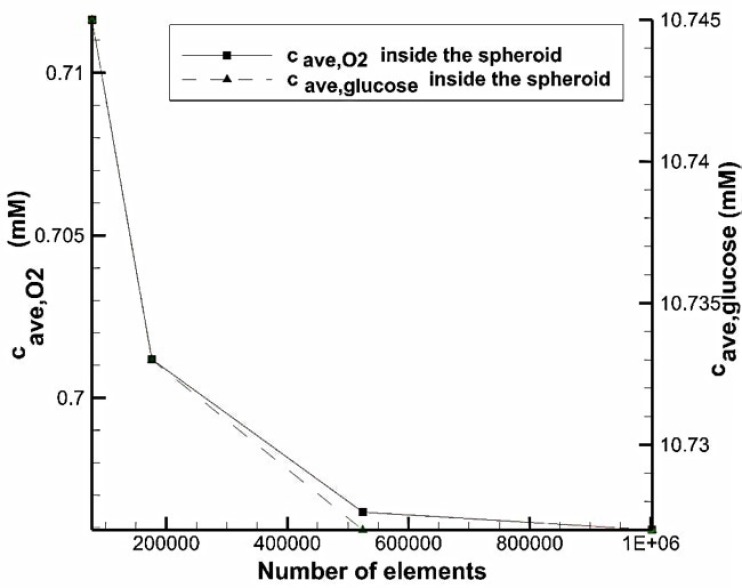
Grid independency study.

To show the independency of results from the number of computational elements, for different number of cells, average oxygen and glucose concentration in the whole volume of the spheroid in the system with U-shaped barrier were measured (Figure 3). The volume of the spheroid is equal to the volume of the toroid with r=80 μm and $\frac{R}{r}=2$. We observed that increasing the number of elements makes the data to converge to an approximately constant value. With a total number of cells of 524,433, the error is less than 0.5% compared to finer mesh.

**Figure 4:**
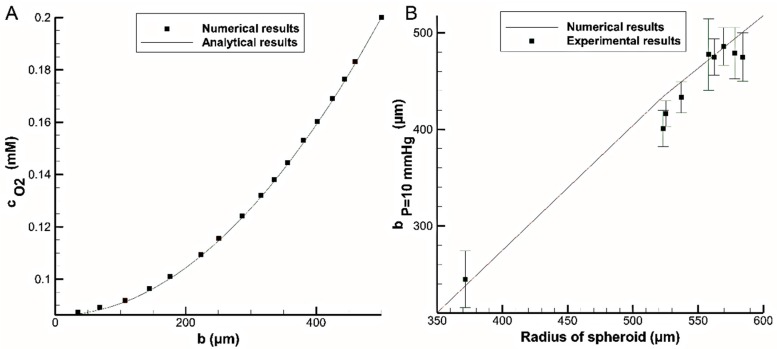
Comparison between the oxygen distribution obtained from present numerical results with: (**A**) analytical solution, Equation (5), ($R_{s}$ = 500 μm, $c_{O_{2},surface}=$ 0.2 mM and A = 0.005 $\frac{\mathrm{mol}}{m^{3}\cdot s}$) and (**B**) experimental data conducted by Grimes et al. [34] ($c_{O_{2},surface}=0.1322$ mM).

Grimes et al. [34] studied oxygen distribution in the tumor spheroids both experimentally and analytically. The model was one-dimensional and considered a constant reaction rate. The results showed that hypoxic and necrotic zones become larger by increasing the spheroid diameter. The numerical method in this study was first compared to the analytical solution for a simple sphere with a diameter of 500 μm, surface concentration of 0.2 mM and constant reaction rate of 0.005 $\frac{mol}{m^{3}\cdot s}$. The oxygen concentration distribution can be analytically obtained as follows [34]:

(5) $$c=\frac{A}{6D_{O_{2}-CT}}\left(b^{2}-R_{s}^{2}\right)+c_{surface}$$

where *A*, $D_{O_{2}-CT}$, *b*, $R_{s}$ and $c_{surface}$ are reaction rate, diffusion coefficient of $O_{2}$ through cancerous tissue, the radius, the spheroid radius and the concentration at the surface of spheroid, respectively. Figure 4A compares the oxygen concentration distributions obtained with the analytical solution (Equation (5)) and the numerical simulation of this study. The data showed a very good agreement between analytical and numerical simulations with a maximum error about 1%.

Subsequently, we compare our results with the experimental data obtained by Grimes et al. [34] using the same reaction rate for all the simulations. According to that experiment, liquid overlay method was used to grow spheroid multicellular aggregate from human colorectal carcinoma cells. Nine spheroids with different diamters were stained and the culture medium around each spheroid was changed every two days for a total duaration of 17 days. To determine the necrotic radius for each spheroid, the oxygen partial pressure was measured in 360 different regions. For different spheroid radiuses, a radius with an oxygen partial pressure of 10 mmHg ($b_{p=10\;\mathrm{mmHg}}$) was reported (corresponding to hypoxic region). The standard deviation was found to be 10% of the mean value for each reported data set. According to Henry’s law, the hypoxic oxygen partial pressure corresponds to a concentration of $c_{O_{2}}=0.01322\;\mathrm{mM}$. Figure 4B compares the values of $b_{p=10\;\mathrm{mmHg}}$ obtained from the experiment with those obtained from numerical simulation. With a maximum error of 8.5%, the data are in good agreement with the experimental results.

## 3. Results and Discussion 

For all simulations, a spheroid with equal volume to the toroid is used for the comparison ($R_{s}={\left(\frac{3}{2}r^{2}R\right)}^{\frac{1}{3}}$). The height of the barrier is considered as a variable. The results are shown and discussed in the subsections. 

### 3.1. Comparison between U-Shaped Barrier and Microwell

**Figure 5:**
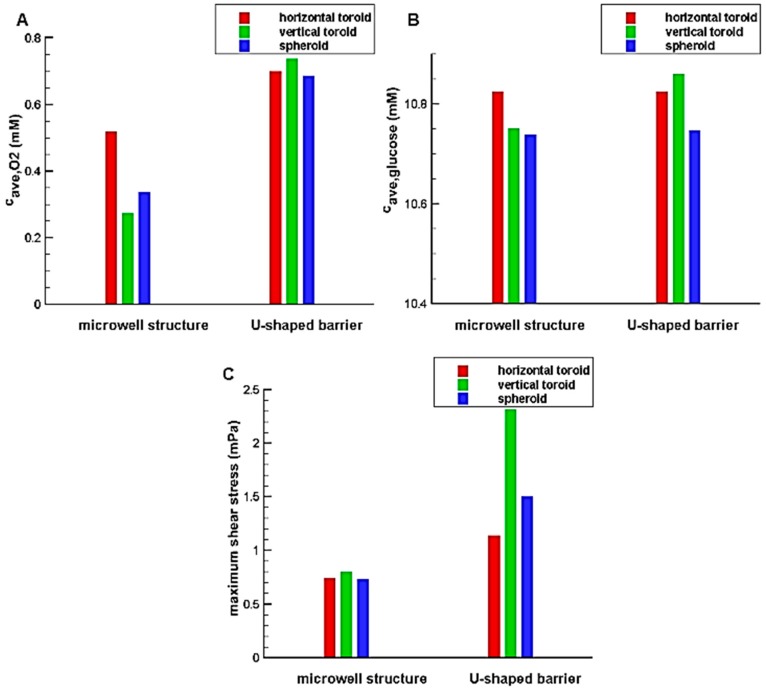
The effect of trap type on: (**A**) oxygen average concentration inside the aggregate; (**B**) glucose average concentration inside the aggregate; (**C**) maximum shear stress on the aggregate.

The simulations are done to find the effect of trap shapes on the oxygen and glucose concentration distributions. As explained, two type of traps, i.e., U-shaped barrier and microwell structure are considered. The toroid has the minor radius of r=80 μm and the aspect ratio (the ratio of major radius to minor radius of the toroid) of $\frac{R}{r}=2$ and the trap (U-shaped barrier and microwell) heights are chosen in a way that completely cover the aggregate. As mentioned before, the spheroid has the volume equal to that of the toroid. The results are shown in Figure 5.

The results illustrate that the system with U-shaped barrier provides higher concentration value for oxygen and glucose in comparison with the system with microwell; so such system can avoid occurring hypoxia and necrosis through the aggregates. The reason is that the aggregated cells in the microwell are far away from the feeding supply, i.e., main stream of the culture medium. In fact, the main stream of the culture medium exchanges oxygen and nutrients mainly due to convection. Although, the culture medium is in contact with the aggregate trapped in the microwell, it has no significant velocity and so the convection effect is less pronounced. However, the cells are directly exposed to the flow and fed for the system with a U-shaped barrier. The difference between the results of two traps for oxygen is greater than those for glucose. This is mainly due to the oxygen permeability of PDMS used in the U-shaped barrier, which improves the oxygen concentration distribution inside the aggregate. Being in direct contact with the flow also increases the shear stress in these systems. According to Figure 5C, the maximum values of shear stress in the U-shaped barrier are far more than those in the microwell structure. Depending on cell type and flow pattern, Shemesh et al. listed various range of allowable shear stress for flow in microdevices [35]. For example, shear rate in a range of 0.25–3 dyn/cm^2^ was reported to be safe for cancer cell culture under continuous fluid flow [35]. Accordingly, for all the cases investigated in this study, the maximum shear stress is in a reasonable and non-injurious range.

In the system containing U-shaped barrier due to the more convection effect, the aggregates with greater heights experience high levels of oxygen and glucose concentration distribution and shear stress. However, the reverse is true in the microfluidic system with microwell structure. This is because the higher aggregate height means a more average distance of the cells from the flow. As a result, it could be expected that independent of the height of the aggregate, increasing the height of the microwell reduces the concentration of oxygen and glucose as well as the shear stress. Therefore, only the effect of the height of the U-shaped barrier is investigated in this research.

### 3.2. The Influence of the Height of a Microfluidic System with a U-Shaped Barrier

**Figure 6:**
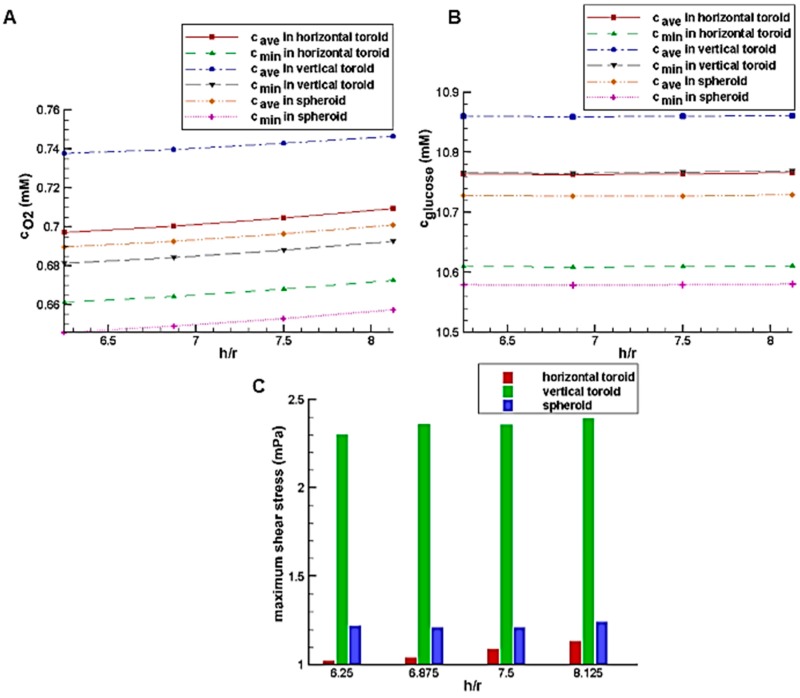
The effect of the relative trap height (*h*/*r* = the ratio of the trap height to the toroid minor radius) on: (**A**) glucose average concentration inside the aggregates; (**B**) average oxygen concentration inside the aggregates; (**C**) maximum shear stress on the aggregates.

The simulations in terms of various U-shaped barrier heights are performed for a toroid with the minor radius of r=80 μm and the aspect ratio of $\frac{R}{r}=2$ as well as for the spheroid with the equal volume. Figure 6A,B shows the effect of the trap height on the glucose and oxygen average concentration inside the aggregate, respectively. Figure 6A shows that by increasing the height of the trap, the average concentration of oxygen increases slowly. However, the dependency of the glucose concentration on the trap height is almost negligible (Figure 6B). Compared to the horizontal toroids, the spheroids have access to the main stream of the culture medium with a higher velocity which mainly delivers both oxygen and nutrients (here, glucose) to the tissue by convection process (the term $\vec{V}\cdot \vec{𝛻}C$ in Equation (3)). On the other hands, these concentrations of oxygen and glucose from the culture medium will be available to cells through pure diffusion (Equation (3c)). This diffusion distance is higher in the spheroids compared to that in the horizontal toroid. However, the vertical toroids do not have the limitation of the spheroids (i.e., high diffusion distance) as well as that of the horizontal toroids (i.e., low convection effect). That is, the vertical toroids are exposed to the high-velocity flow of the culture medium, and their diffusion distance is small enough. Therefore, they have the most level of oxygen and glucose.

**Figure 7:**
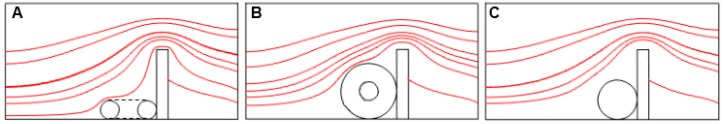
Streamline around: (**A**) the horizontal toroid; (**B**) the vertical toroid; (**C**) the spheroid at middle *x*-*z* plane for *r* = 80 μm, *R*/*r* = 2 and *h*/*r* = 7.5.

Figure 6C shows the effect of the trap height on the maximum shear stress on the aggregate surface. According to the figure, the trap height in this range has a low effect on the shear stress on the aggregate too. By approaching the higher velocity areas, due to no-slip condition on the aggregate, the velocity gradient as well as the shear stress increases. Figure 7 shows the deviation of the flow streamlines in the presence of the aggregates, which indicates the sharp velocity gradients. The more significant deviation of the streamlines for the vertical toroid and then for the spheroid case justifies the results of Figure 6C. Thus, the shear stress on the surface of the vertical toroid is more than that of the spheroid. Also, the shear stress on the surface of the spheroid is more than that of the horizontal toroid. Since the shear stress on the vertical toroid surface is the highest, when using this orientation of the toroid, the allowable shear stress should be taken into account. Because the shear stress is small for the horizontal toroid, higher values of flow rate of the culture medium can be used for the cells in the shape of a horizontal toroid.

Due to the higher level of oxygen and glucose in the system with the U-shaped barrier as well as normal range of shear stress, this system is chosen and used for the next simulations.

### 3.3. Comparison between Toroid and Spheroid in a System with a U-Shaped Barrier 

To investigate the effects of the toroid aspect ratio and minor radius on the glucose and oxygen concentration distribution, the simulations are done in a system with a U-shaped barrier with the fixed height of 600 μm (*h*/*r* = 7.5). As explained before, the U-shaped trap height has no significant influence on the oxygen and glucose concentration distributions inside the aggregate as well as shear stress on its surface.

**Figure 8:**
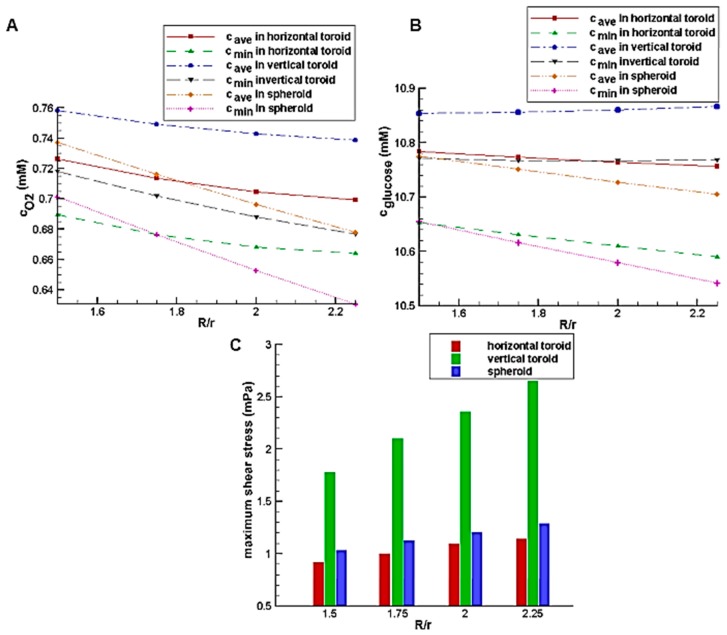
The effect of toroid aspect ratio on: (**A**) glucose average concentration inside the aggregate; (**B**) oxygen average concentration inside the aggregate; (**C**) maximum shear stress on the aggregate surface.

Figure 8A,B show the effect of the toroid aspect ratio with the constant toroid minor radius of 80 μm on the glucose and oxygen concentration distributions, respectively. In low aspect ratio, due to the small size of spheroid and toroid and short diffusion distance as well as the better access of the spheroid to the higher velocity fluid flow because of its height, the average concentrations of glucose and oxygen in the spheroid is higher than those of the horizontal toroid. However, with an increase of aspect ratio and an increase of diffusion distance in the spheroid, despite the higher external concentrations (the concentration of the culture medium covered the aggregate), oxygen and nutrient deficiency areas are created. It results in the higher levels of oxygen and nutrients inside the horizontal toroid in comparison with the spheroid. The minimum concentration, which can indicate the presence or absence of hypoxia and necrosis, has a similar trend. Because of the lower diffusion coefficient of glucose than oxygen, the first case results (lower aspect ratio) is not very noticeable. In all aspect ratio, the vertical toroid has the highest oxygen and glucose concentration with a significant difference to the others. The reason for this, as stated, is the lack of restrictions of the other two aggregate (greater diffusion distance in spheroid and lower convection effect in horizontal toroid).

The effects of the aspect ratio on shear stress are shown in Figure 8C. By increasing the toroid aspect ratio, the height of all aggregates is enhanced, so the streamline deviation and also the maximum shear stress on the aggregate surface are increased.

**Figure 9:**
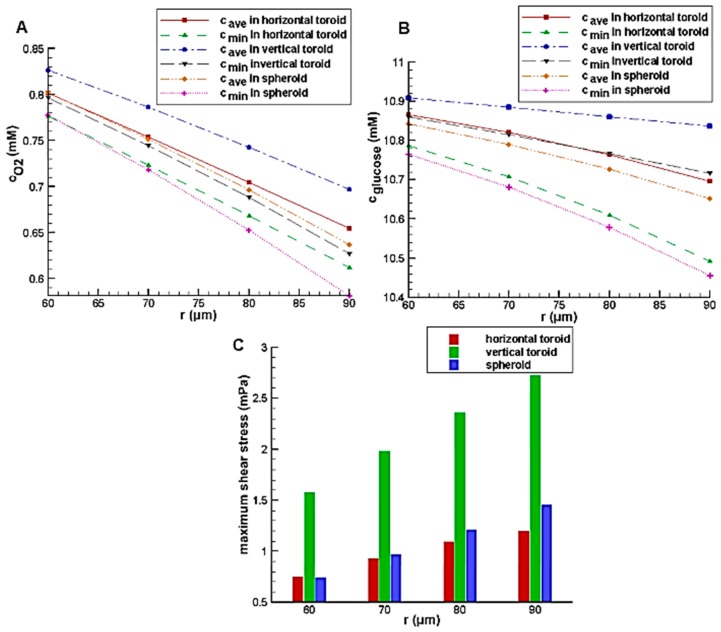
The effect of toroid minor radius on: (**A**) glucose average concentration inside the aggregate; (**B**) average oxygen concentration inside the aggregate; (**C**) maximum shear stress on the aggregate surface.

Figure 9 shows the effect of the minor radius on the glucose and oxygen concentration and maximum shear stress on the aggregate surface with the constant toroid aspect ratio of 2. Similar to aspect ratio, increasing the minor radius leads to an increase in the aggregate volume. This will increase its height and diffusion distance, so the same trend is observed.

**Figure 10:**
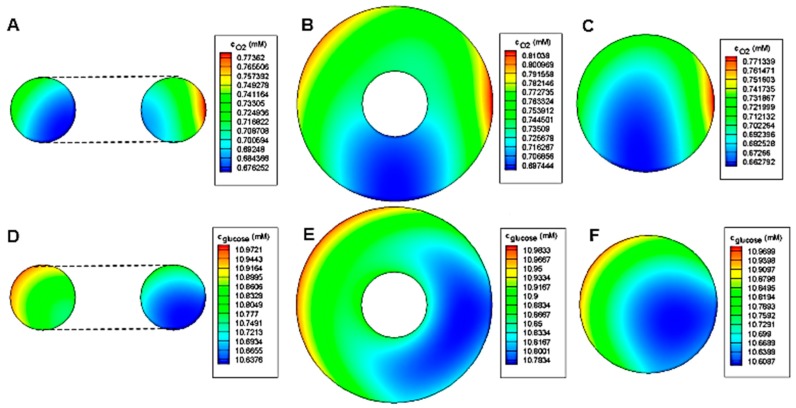
(**A**–**C**) Oxygen concentration distribution and (**D**–**F**) Glucose concentration distribution in horizontal and vertical toroid and spheroid at middle *x*-*z* plane for *r* = 80 μm, *R*/*r* = 2 and *h*/*r* = 7.5.

The concentration distribution inside toroid and spheroid for *r* = 80 μm, *R*/*r* = 2 and *h*/*r* = 7.5 is shown in Figure 10. As expected, the minimum concentration of both oxygen and glucose belongs to the spheroid case. Due to the oxygen permeability of PDMS, the oxygen concentration distribution is relatively symmetrical to the centerline. However, for glucose, the deficiency area tends to the side of the barrier because of the impermeability of PDMS to glucose. 

On a non-adherent surface, toroid tissue exhibits radial contraction and cell migration to close the inner ring over time [16,36]. The results of this paper can shed light on toroid closure mechanism. Toroid tissue closure processes can be affected by the efficiency of metabolite exchange of both oxygen and glucose. This depends on the sufficiency of tissue perfusion. Vertically positioned toroid tissue can be exposed to laminar flow perfusion. By this, the medium passes through the toroid opening. This may also improve metabolites diffusion from the opening. Thus, the necrotic effect can be reduced. As a result, tissue metabolism will be raised to enhance toroid contraction. However, fluid motion through the inner opening generates shear stress. This stress may act in the radial direction and influence the contraction process. Thus, toroid tissue must compensate the circumferential stress to deform. In this case, toroid tissue can exhibit elastic behavior subject to tunable fluid phase motion. This strategy mimics physiological shear stress in vitro and may resemble as a simple tissue structured with lumen.

## 4. Conclusions

In this study, we first illustrated the difference in cell density for the formation of 3D multicellular aggregates of toroid and spheroid. We then employed numerical solutions with finite element approach to systematically compare toroid and spheroid cell culture. To this aim, shear stress, as well as oxygen and glucose concentration distributions, were obtained inside the aggregates in the forms of spheroid and toroid in a microfluidic system containing either a U-shaped barrier or a microwell structure. Continuum and momentum equations were solved for culture medium flow. Convection-diffusion equations in two regions of the culture medium and the cellular aggregate were solved to find the oxygen and glucose concentrations. Since most microfluidic devices are fabricated from PDMS (which is permeable to oxygen), convection-diffusion equations were also solved in an additional region of the oxygen-permeable wall to find the concentration of oxygen. In these simulations, two orientations for the toroid, i.e., horizontal and vertical ones, were investigated and compared.

The results showed that oxygen and glucose concentration inside the aggregate and maximum shear stress on its surface in a system with U-shaped barrier is higher than those in the microwell structure. It was also found that the effect of the trap height was almost negligible in the system with U-shaped barrier. The concentration level in spheroid and horizontal toroid depends on their volume. For the small aggregate, spheroid had higher oxygen and glucose concentrations. However, these concentrations were higher in large cellular agregates in the form of horizontal toroid. For all the cases, the maximum shear stress on the spheroid was higher than that of the horizontal toroid due to the more deviation of the streamlines. The orientation of the toroid in the trap affects the glucose and oxygen concentration distribution inside the aggregate and also the maximum shear stress on its surface. The results revealed that among all the cases, vertical toroid has the highest values of shear stress as well as the oxygen and glucose concentrations. These results can be used as guidelines to select and design the optimum microfluidic bioreactor when making a trade-off between the shear stress and oxygen/nutrient concentrations.

## Figures and Tables

**Table 1:** The effect of margin distance on the simulation results for a horizontal toroid with *r* = 80 μm, *R*/*r* = 2 and *h*/*r* = 7.5.

Parameters	$c_{ave,O_{2}}\left(mM\right)$	$c_{ave,glucose}\left(mM\right)$	*τ_max_* (mPa)
Margin = 1 μm	0.69649	10.727	0.0012088
Margin = 0	0.69687	10.726	0.0012102
Difference	0.05%	0.009%	0.1%

**Table 2:** The simulation properties [22].

Parameters	Values	Descriptions
$\rho_{H_{2}O-37\;℃}$	993.3 $\frac{\mathrm{kg}}{m^{3}}$	Density of water at 37 °C
$\mu_{H_{2}O-37\;℃}$	0.000692 Pa·s	Viscosity of water at 37 °C
$D_{O_{2}-H_{2}O}$	$2.6\times 10^{-9}\;\frac{m^{2}}{s}$	Diffusion coefficient of $O_{2}$ through $H_{2}O$
$D_{O_{2}-PDMS}$	$3.4\times 10^{-9}\;\frac{m^{2}}{s}$	Diffusion coefficient of $O_{2}$ through PDMS
$D_{O_{2}-CT}$	$1.83\times 10^{-9}\;\frac{m^{2}}{s}$	Diffusion coefficient of $O_{2}$ through cancerous tissue
$D_{glucose-H_{2}O}$	$9.27\times 10^{-10}\;\frac{m^{2}}{s}$	Diffusion coefficient of glucose through $H_{2}O$
$D_{glucose-CT}$	$2.7\times 10^{-10}\;\frac{m^{2}}{s}$	Diffusion coefficient of glucose through cancerous tissue
$S_{O_{2}-PDMS\;vs\;H_{2}O}$	6	Solubility of $O_{2}$. in PDMS vs $H_{2}O$
$S_{O_{2}-CT\;vs\;H_{2}O}$	4.81	Solubility of $O_{2}$ in cancerous tissue vs $H_{2}O$
$S_{glucose-CT\;vs\;H_{2}O}$	1	Solubility of glucose in cancerous tissue vs $H_{2}O$
$V_{max.\;O_{2}}$	0.0203 $\frac{\mathrm{mM}}{s}$	Maximum reaction rate of $O_{2}$
$V_{max.glucose}$	0.01076 $\frac{\mathrm{mM}}{s}$	Maximum reaction rate of glucose
$K_{m.\;O_{2}}$	0.00463 mM	Michaelis constant of $O_{2}$
$K_{m.glucose}$	0.04 mM	Michaelis constant of glucose
